# A Treatise on the Diseases of the Air Passages

**Published:** 1847-12

**Authors:** 


### ARTICLE XX.

*A Treatise on the Diseases of the Air Passages;* comprising an inquiry into the history, pathology, causes and treatment of those affections of the throat, called bronchitis, chronic laryngitis, clergyman's sore throat, etc. etc. By Horace Green, M. D., formerly president and professor of the theory and practice of medicine in the Castleton Medical College, &c. &c. New York and London. 1846. pp. 272. 8vo.

We have omitted to notice this work which has been for more than a year before the public, until the present time, being, in a great measure, influenced by the notices of the medical press, which not being, in several instances, favorable, prevented us from giving it an examination. But having recently perused it, we were favorably disappointed in regard to its merits, and think it deserving of the careful attention of practical physicians.

It is, so to speak, a book of one idea; the affections enumerated in the title page. being treated with cauterization of the orifice and internal surface of the larynx with nit. arg., in dose of about forty grains of the pure crystals to the ounce of water. The simple mention of such an operation is sufficient to excite the surprise of those accustomed to think that the slightest contact of water even, will excite violent spasm and closure of the glottis, and it requires some reflection to induce us to try it, and actual experience to convince us that the application of the above named solution is attended with and followed by less irritation than is the result of the contact of the simplest fluid. Such, however, seems to be the fact, and having used a solution of half the above named strength to the orifice or internal surface of the larynx, in three instances, we can testify that it was followed by no considerable irritation.

But if safe, is it useful? In answer to this question, Dr. Green gives the reports of a large number of cases treated in this way, which go far to establish the affirmative of the question, without, however, specifying the circumstances in which it is likely to be useful, or indicating any circumstances in which its use might be followed by inconvenient effects.

The following extract will show the manner in which the application is to be made.

"*Method of applying the solution.*—In the treatment of laryngeal disease, by the direct application of the nitrate of silver, to the diseased surface, I have employed, ordinarily, a solution of this substance, of the strength of from two to four scruples of the nitrate, to an ounce of distilled water. When, however, there are found extensive ulcerations of the epiglottis, or, about the opening of the larynx—ulcerations which it is desirable to arrest at once, I have not hesitated to apply directly, to the diseased parts, a solution of double the strength of the last named. But one or two applications only, of a medicine of this power should be made, at one time; ordinarily, however extensive the lesions may be, it will not be necessary to employ a solution of greater strength than one composed of four scruples of the salt, to an ounce of water. On the other hand, it has been found, that one of less strength than of from forty to fifty grains of the nitrate to the ounce of fluid, will have but little effect upon a diseased mucous surface, where ulcerations exist.

"In cases in which it becomes necessary to cauterize the interior of the laryngeal cavity, the aperture of the glottis should not be passed at once; the part should be *educated*, by applying the solution daily, for several days, to the faucial and pharyngeal region; to the epiglottis, and about the opening of the glottis.

"Proceeding in this manner, that exquisite sensibility which belongs to the lips of the glottis, is, in a good degree, overcome, and the instrument may then be passed into the larynx, without producing half the amount of that irritation, which its introduction below the epiglottis would have awakened at first.

"The instrument which I have always employed for making direct medicinal applications into the cavity of the larynx, is one composed of whalebone about ten inches in length, (with or without the handle as represented in the plate) curved at one end, to which is securely attached a small, round piece of fine sponge.

"The extent to which the rod is bent, must be varied according to circumstances, for the opening of the glottis is situated much deeper in some throats, than in others; but the curve which I have found suited to the greatest number of cases, is one which will form the arc of one quarter of a circle, whose diameter is four inches. (See Plate VII. Fig. I.)

"The instrument being prepared, and the patient's mouth opened wide, and the tongue depressed, the sponge is dipped into the solution to be applied, and being carried over the top of the epiglottis, and on the laryngeal face of this cartilage, is suddenly pressed downwards and forwards, through the aperture of the glottis into the laryngeal cavity.

"This operation is followed by a momentary spasm of the glottis, by which the fluid is discharged from the sponge, and is brought into immediate contact with the diseased surface.

"Every physician who has been present when the operation has been performed, (and a large number have witnessed it from time to time,) has manifested much suprise on observing how little irritation has been produced by the introduction of the sponge."

Time and experience will be required to determine the value of the treatment, for if it has been proposed and used by Trousseau and Belloc, it has not been so generally applied as to have its value properly tested. In the mean time, it seems to us highly probable that its use in many cases of chronic inflammation of the throat, is worthy of a careful trial. D. B.

